# Preoperative ^68^Ga-DOTA-Somatostatin Analog-PET/CT Hybrid Imaging Increases Detection Rate of Intra-abdominal Small Intestinal Neuroendocrine Tumor Lesions

**DOI:** 10.1007/s00268-017-4364-1

**Published:** 2017-11-20

**Authors:** Olov Norlén, Harald Montan, Per Hellman, Peter Stålberg, Anders Sundin

**Affiliations:** 1 Department of Radiology and Molecular Imaging, Institute of Surgical Sciences, Uppsala University Hospital, Uppsala University, 751 85 Uppsala, Sweden; 2 Department of Surgery, Institute of Surgical Sciences, Uppsala University Hospital, Uppsala University, Uppsala, Sweden

## Abstract

**Background:**

Small intestinal neuroendocrine tumors (SI-NETs) are the most common form of neoplasm in the small bowel. Radiological identification of primary tumors (PT), which may be multiple, is difficult, and therefore palpation of the entire small bowel is routinely performed during laparotomy. The aim was to determine detection rates of PT and peritoneal carcinomatosis (PC) with ^68^Ga-DOTATOC/TATE-PET/CT in comparison with i.v. contrast-enhanced computed tomography (CE-CT) and thus to clarify whether modern functional imaging can mitigate the need for palpation of bowel during surgery enabling oncologically adequate laparoscopic resection.

**Methods:**

A total of 28 patients with SI-NET who preoperatively underwent both ^68^Ga-DOTATOC/TATE-PET/CT and CE-CT were included. The detection rates of PT and PC for PET/CT and CE-CT were compared to the findings in the surgical and histopathological reports. Appropriate statistical tests were used, and significance was set to *p* < 0.05.

**Results:**

Out of 82 PT, 43 PT were not detected by any imaging modality. More PT lesions were detected with PET/CT (*n* = 39 [47.5%]) than with CE-CT (*n* = 10 [12.2%], *p* < 0.001). Also, PET/CT identified significantly more PC lesions than CE-CT (78 and 38%, *p* = 0.004, respectively).

**Conclusion:**

PET/CT detected more PT and PC lesions than CE-CT. Some PTs and PC lesions were only detected by one of the modalities, and CT performed in conjunction with PET/CT should therefore be performed as a fully diagnostic CE-CT for optimal results. Palpation of the small bowel remains crucial during surgery in these patients because several PTs escaped detection by both PET/CT and CE-CT.

## Introduction

Neuroendocrine tumors (NETs) comprise a heterogeneous group of rare [1, 2] epithelial neoplasms of neuroendocrine differentiation [3, 4]. They emanate from neuroendocrine cells of the diffuse neuroendocrine system and are characterized by their ability to produce peptides that can cause characteristic hormonal syndromes [2, 5–9]. NETs originating in the gastrointestinal tract, including the pancreas, are denoted gastroentero-pancreatic (GEP-NETs [2], of which small intestinal NETs (SI-NETs) are the most common [2, 10, 11]. Most SI-NETs express somatostatin receptors on the cell surfaces, which is used for both imaging and treatment [12]. The primary tumor in the small bowel is usually quite small, but in up to 35% of cases there may be multiple primary tumors in the bowel wall [13]. Loco-regional disease with metastatic lymph nodes (LNM) in the mesentery is very common and present in over 90% of patients at diagnosis [13]. In these patients, surgical resection may be curative although the risk of long-term relapse is common based on historical data [13]. The most common distant metastases are hepatic, followed by peritoneal carcinomatosis (PC) [13]. In disseminated disease, first-line systemic treatment of SI-NET is long acting somatostatin analogs (SSAs) which prolongs time to progression [14]. Moreover, resectable hepatic metastases may be treated by liver resection alone and/or with radiofrequency/microwave ablation, both to decrease local and hormone-related symptoms, and in an effort to prolong survival, although a survival benefit is not unequivocally proven [15]. In patients with non-resectable, disseminated liver metastases, loco-regional treatment with trans-arterial embolization (TAC) and trans-arterial chemoembolization (TACE) or peptide receptor radionuclide therapy (PRRT) is available. PRRT was in fact recently shown to improve both PFS and OS in a randomized study of progressive SI-NET [16].

Before, during and after treatment, adequate radiological staging is imperative to choose the optimal treatment and surveillance strategy [12]. Conventional morphological imaging methods (computed tomography (CT) or magnetic resonance imaging (MRI)) are considered mandatory for NET staging [12, 17, 18]. The sensitivity of CT and MRI is unfortunately poor for detection of primary small bowel tumors and PC, similarly to the situation in other diagnoses such as PC in colorectal cancer. Somatostatin receptor scintigraphy has been used for decades to image and survey SI-NET; however, currently positron emission tomography (PET) and concomitant CT (PET/CT) with ^68^Ga-DOTA-somatostatin analogs (SSAs) are preferred where available, because of the considerably better imaging yield for distant metastases [12]. To our knowledge, functional imaging of PTs and PC has not been studied in SI-NET patients in particular.

In our center, laparotomy with careful examination of the abdominal cavity, palpation of the small bowel and liver, constitutes the gold standard for staging the abdominal tumor load in patients with SI-NETs and to ensure adequate oncological resection when possible. Others have, in light of the increasing use of laparoscopic surgery in colorectal cancer, applied laparoscopic surgery in SI-NET patients; however, this method mitigates the possibility to palpate the small bowel and multiple primary tumors may be overlooked and left in situ.

The aim of this study was to investigate the sensitivity of ^8^Ga-DOTA-SSA-PET/CT and CE-CT to detect SI-NET primary tumors and intra-abdominal metastases in order to conclude whether preoperative imaging with PET/CT and/or i.v. contrast-enhanced CT (CE-CT) might be accurate enough to enable laparoscopic surgery in these patients without the risk of leaving primary tumors in situ.

This study was therefore designed to determine the preoperative detection rates of PT and PC by ^68^Ga-DOTA-SSA-PET/CT in comparison with CE-CT to clarify whether modern functional hybrid imaging can make the need for palpation of the bowel during surgery obsolete and thus enable oncologically adequate laparoscopic SI-NET resection. Also, correlation between radiology and operative findings with current PC classification systems was investigated.

## Materials and methods

### Patients

In this retrospective clinical observation study, the study population was identified through the hospital data service unit by retrieving clinical data for all patients treated at or cared for in Uppsala University Hospital, during the time period January 01, 2011, to October 25, 2016, with the ICD-10 diagnosis “C17.9 Non-specified location of malignant tumor in the small intestine” and a histopathologically proven SI-NETs diagnosis. Initially, 220 patients were included. After exclusion of patients who had not undergone preoperative ^68^Ga-DOTA-SSA-PET/CT (*n* = 160) or intravenously contrast-enhanced triple-phase CT (*n* = 4), who underwent imaging more than 12 months before surgery (*n* = 3), who did not undergo surgery (*n* = 20) and those for whom the original surgical report could not be retrieved (*n* = 5), ultimately 28 patients (15 males, 13 females), median age 66.5 (range 24–78), were included in the study (Table 1). The study was approved by the local ethics committee.

**Table 1:** Clinical patient data, tumor findings, TNM stage and tumor proliferation index

Patient no.	Age (years)^a^	Sex M/F	Primaries OP/PAD	T	N	M	TNM stage (all data)^b^	KI-67 (%)
1	59	M	1	T4	N1	Liver, PC	T4N1M1	7
2	71	F	3	T3	N1	Liver, bone, PC	T3N1M1	15
3	78	M	1	T4	N1	Liver	T4N1M1	<1
4	24	F	1	T3	N1	Liver	T3N1M1	2
5	59	M	2	T3	N1	Liver, extra-abd. lgll.	T3N1M1	2
6	73	M	3	T3	N1	0	T3N1M0	<1
7	63	M	1	T2	N1	0	T2N1M0	1
8	68	F	1	T4	N1	Liver, ovary, PC	T4N1M1	3
9	78	F	1	T4	N1	Liver, ovary, appendix PC	T4N1M1	5
10	66	F	2	T4	N1	Liver	T4N1M1	3
11	69	M	6	T3	N1	0	T3N1M0	<1
12	64	M	4	T3	N1	0	T3N1M0	<1
13	74	M	3	T3	N1	0	T3N1M0	1
14	76	F	1	T3	N1	PC	T3N1M1	<1
15	47	M	1	T3	N1	0	T3N1M0	<1
16	72	M	2	T3	N1	0	T3N1M0	3
17	57	M	10	T4	N1	Liver, PC	T4N1M1	2
18	72	M	17	T3	N1	0	T3N1M0	<1
19	59	M	1	T3	N1	Liver	T3N1M1	2
20	61	F	4	T4	N1	Liver, PC	T4N1M1	4
21	67	M	3	T4	N1	Liver	T4N1M1	<1
22	54	F	2	T3	N1	0	T3N1M0	<2
23	73	F	6	T4	N1	0	T4N1M0	3.4
24	74	F	2	T3	N1	0	T3N1M0	2–3
25	59	F	1	T4	N1	Liver, PC	T4N1M1	0.5
26	70	F	1	T2	N1	0	T2N1M0	<2
27	63	M	1	T4	N1	Liver	T4N1M1	1
28	40	F	1	T3	N1	Liver	T3N1M1	1

^a^Age at the time of surgery

^b^Data from imaging, surgery and histopathology

The tumors were classified according to the ENETS 2010 histopathological grading system for GEP-NETs based on their proliferation (ki-67 index) (2) (Table 1), and tumor staging was performed by using the WHO 2010 TNM-grading and staging system according to WHO and ENETS consensus (2).

### Preoperative imaging and image analysis

PET/CT was performed with ^68^Ga-DOTA-D-Phe^1^-Tyr^3^-octreotide (^68^Ga-DOTATOC) in 18 patients and ^68^Ga-DOTA-D-Phe^1^-Tyr^3^-octreotate (^68^Ga-DOTATATE) in 10 patients. Approximately 2 MBq/kg body weight of ^68^Ga-DOTATOC/TATE was injected as an intravenous bolus, and after 60 min examination was performed on a GE Discovery ST PET/CT scanner (General Electric Health Care, Milwaukee, Mich, USA) and included from the base of the scull to the proximal thighs. A low radiation dose non-contrast-enhanced CT was performed in connection with PET and was used for attenuation correction and anatomical correlation of the PET findings.

Contrast-enhanced CT (CE-CT) was performed according to a clinical standard examination protocol whereby the liver was examined before and during intravenous contrast-enhancement in the late arterial phase, and then, the whole abdomen and pelvis were examined in the venous phase. The original 0.63-mm transversal slices were reformatted as 3-mm images in the transversal, coronal and sagittal planes (multiplanar reformatted images, MPRs).

Two readers, one with basic radiological training and one senior consultant radiologist, analyzed the images separately and then together in consensus. The images were viewed using the hospitals´ picture archiving and communication system (PACS). To exclude image reading bias, the patients’ identities were coded (A01, A02, etc.). The preoperative tumor status (location, extent and lesion sizes) was recorded for CE-CT and PET/CT regarding the primary tumor, regional and distant lymph node metastases, peritoneal carcinomatosis, liver metastases and other distant metastases. On CE-CT, lesion size was measured as the largest transversal diameter. Lesions only visualized by PET but not on CT were not measured. Lymph nodes were on CT characterized as metastases when their diameter was ≥10 mm (short axis) and accumulating the i.v. iodine-based contrast medium similarly to that of the primary tumor and liver metastases. A conglomerate of metastatic lymph nodes was registered as one single node. Contrast-enhancing intraperitoneal lesions were identified as PC if their largest diameter was ≥5 mm. On ^68^Ga-DOTATOC/TATE-PET/CT, any non-physiological focal tracer accumulation higher than the background activity was evaluated as tumor. Peritoneal tracer uptake with a correlating morphological lesion on CT with a largest diameter ≥5 mm was regarded as PC. Further, for both ^68^Ga-DOTA-SSA-PET/CT and CE-CT, in the case of only identifying part of the PC lesions compared to the standard of reference, the method was still interpreted as able to detect the PC lesions on patient basis.

### Standard of reference

The combination of histopathological records and the surgical report of the primary tumor surgery were used as the standard of reference. Similar to the imaging assessment, the patients’ identities were coded (A01, A02, etc.). When the standard of reference did not expressly record the presence of PC, the interpretation was made that the patient had no PC. Moreover, due to the difficulty of knowing whether peritoneal implant in the ovaries originated from hematogenic or peritoneal spread, these cases were interpreted as peritoneal carcinomatosis.

The extent of PC was assessed by using a Gilly Lyon PC classification system [19].

The Gilly Lyon PC classification intra-operatively stratifies PC into five stages and can be applied prospectively and retrospectively, based on surgical reports [19–21], and can additionally be assessed on imaging [18]. The Gilly Lyon PC stages 0–4 are: stage 0: no macroscopic PC findings; stage 1: malignant granulation <5 mm in size localized in one part of the abdomen; stage 2: malignant granulations <5 mm, diffuse localization; stage 3: localized or diffuse malignant granulations 5–20 mm in size; and stage 4: localized or diffuse large malignant masses >20 mm in size.

### Statistical methods

Data were recorded and analyzed by using Microsoft^®^ Excel and IBM^®^ SPSS software. Continuous variables were presented as median and range, and categorical data were expressed as number and percentage. Patient-based and lesion-based detection rates of primary tumors, loco-regional lymph node metastases and peritoneal carcinomatosis were calculated. The sensitivity, specificity and positive predictive values (PPV) for primary tumor and PC detection, as compared to the standard of reference, were calculated in a patient-based and lesion-based analysis for CE–CE and PET/CT. To test differences between groups, McNemar’s test was applied. A *p* <0.005 was considered significant.

## Results

### Tumor grading and tumor staging

There were 19/28 (68%) G1 NETs and 9/28 (32%) G2 NETs with Ki-67 index median 1% (range 1–14%). No G3 tumors (NECs) were found. According to the WHO 2010 TNM-staging, based on the combination of surgical reports, histopathological records and image findings, 11/28 (39%) of the patients were in stage IIIb and 17/28 (61%) in stage IV (Table 1).

### Patient-based analysis

The prevalence of PC in the study population according to reference standard was 29% (8/28 patients). Table 2 demonstrates the patient-based image analysis. Significantly, more patients were diagnosed with primary tumors by ^68^Ga-DOTATOC/TATE-PET/CT, 25/28 (89%) compared to CE-CT 7/28 (25%), *p* < 0.001. Regional lymph node metastases were detected in 100% of patients by both PET/CT and CE-CT. The sensitivity, specificity, positive predictive value (PPV) and negative predictive value (NPV) to detect PC were for ^68^Ga-DOTATOC/TATE-PET/CT 63, 90, 71 and 86%, respectively, and the corresponding figures for CE-CT were 75, 100, 100 and 91%, respectively. PET/CT falsely diagnosed PC in two patients.

**Table 2:** Patient-based imaging findings^a^ as compared to the standard of reference (surgical findings in combination with histopathology)

	^68^Ga-DOTATOC/TATE-PET/CT	CE-CT	Surgery and histopathology
Primary tumors	25 (89)	7 (25)	28 (100)
Regional lymph node metastases	27 (100)	27 (100)	27 (100)
PC	7 (88)	6 (75)	8 (100)

^a^Values are number (%), *PC* peritoneal carcinomatosis

### Lesion-based analysis

**Fig. 1:**
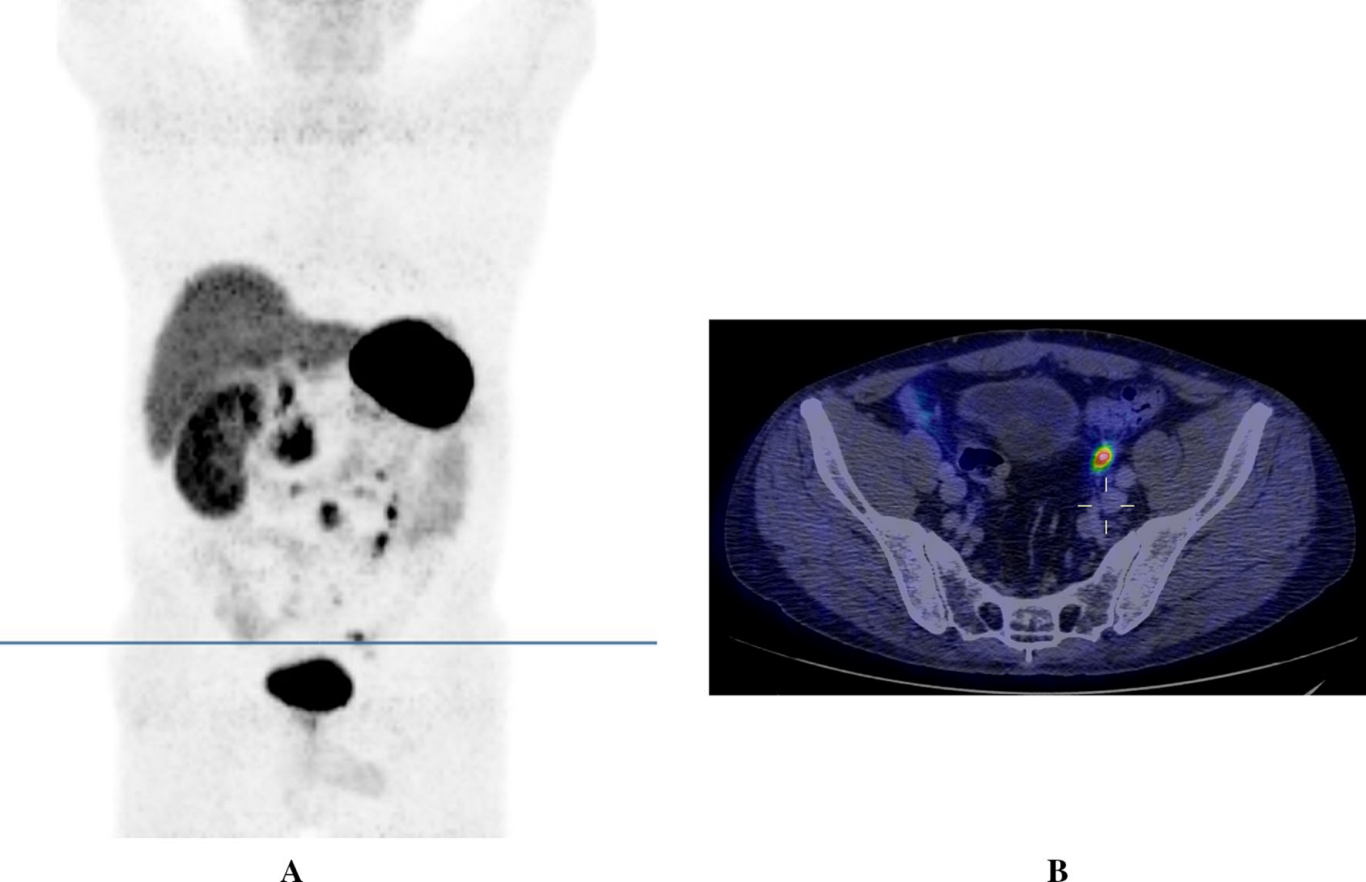
^68^Ga-DOTATOC-PET/CT. 3D PET reconstruction (maximum intensity projection) showing multiple peritoneal carcinomatosis lesions predominately in the left lower part of the abdomen (**a**). The transverse line indicates a peritoneal metastasis in the left pelvis and corresponds to the level of the transverse PET/CT fusion image (**b**) in which the same lesion is shown with a high ^68^Ga-DOTATOC uptake (above the upright post of the cross)

The lesion-based sensitivity and PPV for PC for ^68^Ga-DOTA-SSA-PET/CT were 49 and 63%, respectively, and the corresponding figures for CE-CT were 38 and 100%, respectively (Table 3). Significantly more primary tumors were diagnosed by ^68^Ga-DOTA-SSA-PET/CT, 39/82 (48%) compared to CE-CT, 10/82 (12%), *p* < 0.001 (Table 3). This was also the case for PC where ^68^Ga-DOTA-SSA-PET/CT diagnosed 35/45 (78%) lesions compared to CE-CT 17/45 (38%), *p* = 0.004 (Table 3; Fig. 1).

**Table 3:** Lesion-based imaging findings^a^ as compared to the standard of reference (surgical findings in combination with histopathology)

	^68^Ga-DOTA-SSA-PET/CT	CE-CT	Standard of reference
Primary tumors	39 (48)	10 (12)	82 (100)
PC	35 (78)	17 (38)	45 (100)

^a^Values are number (%), *PC* peritoneal carcinomatosis

In a second viewing session, at which the readers had access to the surgical and histopathological reports and all imaging results, 4 out of 8 PC lesions missed by CE-CT but detected by ^68^Ga-DOTA-SSA-PET/CT, were identified and measured 4–7 mm in size. They were located pararectal and adjacent to the uterus (three lesions in patient 13), adjacent to the sigmoid colon and the right ovary (one lesion in patient 10) and in the ventral aspect of the abdomen between loops of the small intestine (one lesion in patient 25). The three PC lesions that were missed by ^68^Ga-DOTA-SSA-PET/CT but detected by CE-CT still remained undetected by PET/CT in this second viewing session. These three lesions (patient 31) were band-formed 5–7 mm lesions located on the ventral surface of the liver.

Because the absence of PC (true negative lesions) could not unequivocally be established from the surgical records, it was not possible to calculate the specificity and NPVs in the lesion-based analysis. Comparison of imaging sensitivity of loco-regional lymph node metastases on a lesion-basis was not meaningful as many lymph nodes metastases were conglomerates impossible to distinguish with any modality except in the pathological reports.

The image-based classification of PC, according to the Gilly Lyon score, performed by ^68^Ga-DOTATOC/TATE-PET/CT and CE-CT as compared to the standard of reference (surgical findings in combination with histopathology), is shown in Table 4.

**Table 4:** Stratification of the study patients according to the Gilly Lyon score

Score/stage	^68^Ga-DOTA-SSA-PET/CT	CE-CT	Standard of reference
Gilly Lyon Index Stage 0–IV			
0	21 (75)	22 (79)	20 (71)
I	0	0	1(4)
II	0	1 (4)	2 (7)
III	2 (7)	0	1 (4)
IV	5 (18)	5 (18)	4 (14)

Values are number (%)

## Discussion

At the time of initial diagnosis, SI-NETs are frequently disseminated [22]. Distant metastases are most commonly found in the liver (50–60% of patients), peritoneal carcinomatosis (10–33%), extra-abdominal lymph nodes, lungs (3–5%) and bone (1–6%) [23]. In the vast majority of GEP-NET patients with PC, the lesions are multiple and 30–80% of patients with PC present with synchronous liver metastases [19].

From an imaging perspective, larger PC lesions >1 cm are frequently identified on CT/MRI, while sub-centimeter lesions are frequently missed [24]. The correlation between CT findings and the corresponding intraoperative surgical assessment is generally poor [25]. The imaging yield of PET/CT with ^68^Ga-DOTA-somatostatin analogs (most commonly ^68^Ga-DOTATOC, ^68^Ga-DOTATATE and ^68^Ga-DOTANOC) has been shown to surpass that of somatostatin receptor scintigraphy [26] and of CE-CT for many types of NET lesions [27–30] owing to better spatial resolution and tumor-to-normal-tissue-contrast [30]. Any convincing differences in imaging yields between ^68^Ga-DOTA-SSA preparations have not been shown [31, 32].

In the present single-center, retrospective observation study, ^68^Ga-DOTATOC/TATE-PET/CT widely surpassed CE-CT in locating the primary tumor on both a patient basis and a lesion basis. Although ^68^Ga-DOTATOC/TATE-PET/CT performed better than CE-CT to detect the primary tumors, the majority of the primary tumors were nonetheless missed (Table 3). This is most likely explained by the fact that the large majority of primary tumors identified by the surgeon and histopathologically were merely a few millimeters in size, which is generally below the imaging detection level.

For loco-regional lymph node metastases, the two methods showed similarly excellent performance on a patient-basis. As many LNM were conglomerates macroscopically, ^68^Ga-DOTA-SSA-PET/CT and CE-CT could not be used to assess the number of LNM with any certainty.

Furthermore, PET/CT detected more PC lesions than CE-CT, although similar number of patients with PC was diagnosed by both techniques. Nonetheless, CE-CT did find multiple true positive PC lesions in one patient that was missed by PET/CT. Conversely, PET/CT falsely diagnosed PC in two patients with true negative CE-CT finding. There are multiple possible reasons for these two false positive findings; physiological tracer uptake may on PET/CT have been misinterpreted or PC lesions may have been missed during surgery. Our findings suggest that the methods are best used in conjunction and that CT in connection with PET/CT always should be performed as fully diagnostic CE-CT for optimal staging.

The prevalence of PC in the present study was 29% (8/28 patients), similar to what has previously been reported [20]. PC has been shown to represent a significant independent prognostic factor with 5- and 10-year overall survival rates 52 and 32%, and 79 and 54% for patients without or with PC, respectively [33]. The two most commonly used PC classification systems to date are the PCI and Gilly classifications [19, 20]. The PCI classification is complex and not possible to use in the retrospective setting, and therefore, we used the simpler Gilly Lyon classification. As shown in Table 4, the image-based classification was approximately similar for ^68^Ga-DOTATOC/TATE-PET/CT and CE-CT and correlated reasonably well to the standard of reference. The advanced stages (3 and 4) constitute gross macroscopic disease with a less favorable prognosis [33]. Interestingly, it may possible to apply this classification by estimating the score from radiological imaging as shown here and by others [20].

Limitations of this study included a rather small study population (*n* = 28) and possible missing data in surgical and pathology reports. Although the routine since 2010 has been to record the extent of peritoneal carcinomatosis in all surgical reports, some of them differed in their amount of detail, reflecting the varying degree of experience between surgeons and different awareness of importance of lesion documentation. Consequently, image findings may thus have resulted in false positive observations that in fact were true positive. Misinterpretation by the readers of the PET/CT findings is less likely. The anatomical sites of physiological ^68^Ga-DOTATOC/TATE-uptake are well known, including the sometimes-high tracer accumulations in the bowel, which is found in segments of intestine rather than focally. One of the image readers only had basic radiological training at the time of the study, with limited experience in CT and PET/CT reading, but the final imaging results relied on consensus reading together with an experienced specialist (senior consultant).

To conclude, ^68^Ga-DOTA-SSA-PET/CT performs better than CE-CT for primary SI-NET lesion detection. Because the techniques are complementary, CT in conjunction with PET should be performed as CE-CT for optimal imaging yield. A reasonable correlation was found with surgical and histopathological findings for the imaging-based preoperative staging of PC according to the Gilly Lyon classification, with both CE-CT and PET/CT. Despite modern functional and morphological hybrid imaging, many PC lesions and primary SI-NETs were missed and for surgery with curative intent this makes it necessary to palpate the whole small intestine during surgery, consequently making standard laparoscopic resection oncologically inadequate in many SI-NET patients. If the minimally invasive alternative still is pursued, a hand port to facilitate palpation of the bowel is mandatory.
